# The Nuts and Bolts of Food Immunotherapy: The Future of Food Allergy

**DOI:** 10.3390/children5040047

**Published:** 2018-04-04

**Authors:** Sara Anvari, Katherine Anagnostou

**Affiliations:** 1 Department of Pediatrics, Section of Immunology, Allergy and Rheumatology, Texas Children’s Hospital, Houston, TX 77030, USA; Sara.Anvari@bcm.edu; 2 Department of Pediatrics, Section of Immunology, Allergy and Rheumatology, Baylor College of Medicine, Houston, TX 77030, USA

**Keywords:** food allergy, desensitization, oral immunotherapy, sublingual immunotherapy, epicutaneous immunotherapy, children

## Abstract

Food allergies are on the rise and have a major impact on the quality of life of the food allergic child and their family. Currently, the mainstream treatment for food allergies is strict avoidance and elimination of the allergenic food(s) from the patient’s diet in order to prevent an allergic reaction. However, recent advances in research have presented new therapeutic options for food allergic patients that are potentially becoming promising alternatives to traditional treatment. Food immunotherapy is the most popular of these new emerging interventions and has been studied intensively over the last decade for various foods. In this review, we discuss this exciting new development that is aspiring to become part of the mainstream therapy for food allergy.

## 1. Introduction

Food allergies have been on the rise with some reports demonstrating a dramatic 50% increase in food allergies between 1997 and 2011 [1]. It is estimated that 15 million Americans are affected by food allergies with approximately 8% of children being affected [2].

The most common food allergens include milk, egg, soy, wheat, peanut, tree nuts, fish, shellfish, and seeds [3,4]. Cow’s milk, hen’s egg, and peanuts account for the majority of reactions in young children with peanuts accounting for the most common cause of food induced anaphylaxis [4]. Evidence suggests that milk, egg, soy, and wheat immunoglobulin E (IgE)-mediated allergies can be outgrown [5,6,7,8,9,10,11]. However, peanut, tree nut, fish, and shellfish allergies are more persistent and are rarely outgrown. Rates of 20% resolution for peanut allergy and approximately 11% for tree nut allergy are reported in the literature [12]. The current approach to managing IgE-mediated food allergies is strict avoidance of the causal food allergen and immediate treatment of allergic reactions that may occur due to accidental ingestion. It is recommended that all patients have a written food allergy action plan so that symptoms may be treated appropriately and in a timely manner, according to the severity of an allergic reaction.

Food allergy has been postulated to be the result of a loss or delay in oral tolerance development. Du Toit et al. showed that early rather than delayed introduction of food allergens can help prevent the development of food allergies in high risk infants and promote oral tolerance [13,14]. The evidence supported by this study has recently led to updates to national feeding guidelines and the management of food allergy prevention [15]. For individuals where preventative measures are not possible (especially in individuals with an established food allergy), a safe and effective treatment for food allergies is urgently needed.

## 2. Food Immunotherapy

Allergen immunotherapy (AIT) has shown promising results in reducing the risk of life-threatening allergic reactions in individuals accidentally exposed to an allergen [16,17]. Allergen immunotherapy has been approved for the treatment of aeroallergen and insect venom hypersensitivity for many years. Currently, food allergen immunotherapy is under investigative review to determine its efficacy and safety for children with food allergies.

Food immunotherapy can be administered using various routes such as oral (ingested), sublingual (application under the tongue), and epicutaneous (application on the skin). To date, most immunotherapy trials have focused on cow’s milk, hen’s egg, and peanuts; all common childhood allergens. Food allergen immunotherapy aims to increase the threshold of reactivity to the allergenic food by administering gradually increasing doses (oral or sublingual) or fixed doses (epicutaneous) of the relevant allergen daily. This process is known as ‘desensitization’ and requires regular administration of the allergen to be maintained. The ability to tolerate the dose without experiencing an allergic reaction following a prolonged period of treatment discontinuation (typically weeks to months), is known as ‘sustained unresponsiveness’. Immunotherapy studies have demonstrated successful desensitization, however, sustained unresponsiveness is variable depending on the route of immunotherapy. To date, a treatment regimen determining the ideal dose and dosing intervals for food immunotherapy is yet to be determined.

Food allergen immunotherapy is being studied in patients who have a confirmed diagnosis of an established IgE-mediated food allergy. Food allergen immunotherapy protocols vary widely between different studies, but it is clear that they are time-demanding and require many patient visits. Families should also be aware that side effects (usually mild or moderate allergic symptoms) are common with different forms of food immunotherapy. Systemic reactions may also occur as a result of this treatment.

Certain factors increase the risk of reactions and side effects of oral immunotherapy (OIT), therefore, not all patients are good candidates for food immunotherapy. It has been shown that viral infections, menses, and exercise have been associated with reducing the reaction threshold while on OIT [18]. Furthermore, a history of poor compliance with medication, uncontrolled asthma, severe uncontrolled eczema or allergic rhinitis, uncontrolled chronic urticarial, and eosinophilic esophagitis or eosinophilic gastrointestinal diseases are currently contraindications for food allergen immunotherapy in research studies [19,20,21,22]. Therefore, it is important that healthcare professionals review the risks and benefits with the patient and/or family, in order to determine whether an individual would be considered a candidate for immunotherapy.

Food allergen immunotherapy is on route to becoming part of the mainstream management for food allergies in the near future and may present an alternative option to strict avoidance. Successfully desensitized individuals with food allergies will likely be protected from accidental ingestion and may even be able to introduce allergen containing foods into their diet after maintaining treatment for several years [23,24]. This level of protection will help diminish the fear and anxiety resulting from the threat of accidental ingestion and resultant life-threatening reactions. Ultimately, food immunotherapy has the potential to improve the overall quality of life in individuals suffering from food allergies [25].

It is important for healthcare professionals to have an appreciation for these new therapies to identify individuals who could benefit from them. Thus, understanding how each form of immunotherapy works will allow healthcare professionals to properly disseminate the information to their patients and provide a timely referral to an allergist for further evaluation and management (Table 1). At this time, there is wide variation in dosing related to many different research study protocols. There is currently not a unified protocol for food immunotherapy. These differences in dose are not necessarily related to the type of food. There is ongoing discussion on protocol heterogeneity and how to ensure future standardization of food immunotherapy protocols.

## 3. Oral Immunotherapy

OIT begins by administering a very small starting dose of a food allergen (typically 1–25 mg of food protein) usually mixed into a vehicle (i.e., pudding or applesauce). Daily consumption of the dose is advised, followed by an incremental increase in dose every two to three weeks until a predetermined maintenance dose is achieved (usually from 300 mg up to 4000 mg of food protein). The escalation schedule can take approximately 6–12 months to complete depending on the maintenance dose that is to be achieved. The escalation and daily administration of doses results in desensitization to the food protein for the majority of patients (70–90%) [20,23,26,27,28,29]. Currently, protocols require the daily administration of protein, in order to prevent loss of desensitization. Current trials are ongoing for OIT to determine whether alternative dosing intervals can still provide the same level of protection as daily dosing.

Not all patients can tolerate the escalation of the food allergen doses and adverse reactions are commonly seen with OIT. The most common symptoms reported include oral itching and abdominal pain [25]. Abdominal pain and chronic gastrointestinal symptoms are also the most common reasons for discontinuation of OIT and can account for 10–36% withdrawal rates [17]. Anaphylaxis has also been described with both in-hospital and at-home dosing; although severe reactions with OIT are much less common, they do occur and both patients and healthcare professionals need to be prepared for these [18,19,20,21,22,26,27]. To date, no deaths have been reported from OIT. The risk of reactions with OIT is increased when doses are taken irregularly, taken during illness, menses, or before exercise. Reaction risks are also increased when asthma and/or allergic rhinitis symptoms are not well controlled [18,27,29,30]. Cases of eosinophilic esophagitis have been reported in 2.7% of patients undergoing OIT, but have been reported as high as 5.2% [27,31,32,33,34]; Eosinophilic esophagitis is shown to be reversible once OIT treatment is discontinued. It is not yet clear if OIT causes eosinophilic esophagitis or accelerates an underlying disease process, but this is an important consideration prior to participation in food oral immunotherapy. Healthcare providers must undertake a careful and detailed evaluation and provide all the necessary information regarding the risks associated with OIT to patients and families in order to determine whether OIT would be a safe option for each individual participant.

OIT has been investigated for cow’s milk [28,35,36,37], hen’s egg [23,38,39], and peanut [19,20,22,24,29,40,41,42]. Studies have observed that OIT can successfully lead to food allergen desensitization in approximately 70–90% of patients [20,23,26,27,28,29]. The duration of OIT treatment cessation, in order to assess sustained unresponsiveness, is variable across studies and usually between one week to six months [23,24,41,43,44,45,46]. Sustained unresponsiveness, however has only been observed in up to 50% of individuals who were at least school-age or older [23,24,46] but up to 75% in infants and toddlers [40]. This younger age may present the ideal ‘window’ for OIT, although larger studies are needed to confirm this. Unfortunately, the number of studies investigating sustained unresponsiveness (SUR) are few to make general statements about foods, but generally we would expect higher rates of SUR for milk and egg since the natural history of these allergies is very different from peanut. However, results would be highly dependent on the recruited populations, both in terms of age and in terms of persistence and severity of food allergy.

A variety of immune modulatory agents (such as omalizumab) in combination with OIT have also been investigated in small phase 1 and 2 trials. Results suggest that the combination of omaliziumab and OIT results in accelerated desensitization to peanut [47]; however, once omalizumab is discontinued, severity of adverse reactions appears to increase. There has been a recent head to head comparison study of OIT + placebo versus OIT + omalizumab, which has shown that omalizumab improved the efficacy of multi-food OIT and enabled safe and rapid desensitization to multiple foods [48]. Probiotics have also been studied as OIT adjuvants with positive results, but no direct comparison between probiotic alone versus probiotic and OIT has yet been made [44].

Overall, OIT has shown good efficacy in desensitizing food allergic children with an acceptable safety profile [21]. Although OIT is not a cure for food allergies, when administered according to protocol, it can not only provide desensitization to the food allergen, but also lead to improved quality of life for patients and their families by reducing the fear of accidental allergen exposure.

Recent preliminary results from a multicenter phase 2 peanut OIT study report good safety and efficacy from peanut OIT desensitization. The study demonstrated that participants had an 18-fold increase in the amount of tolerated peanut protein, following a six-month build-up to 300 mg of peanut protein followed by a two-week maintenance period [26]. At this time, ongoing phase 3 OIT trials using the same peanut product will further evaluate the safety and efficacy on a prolonged maintenance schedule in a larger cohort of peanut allergic patients.

## 4. Sublingual Immunotherapy

Unlike OIT, sublingual immunotherapy (SLIT) is administered in a liquid form and held under the tongue for a few minutes and then swallowed. The typical starting dose for SLIT is lower than OIT and begins in micrograms, thus gradually elevating the food threshold to reactivity. Doses are escalated in a similar fashion to OIT, but the maintenance dose is only up to 10 mg of food protein, making SLIT less effective when compared to OIT. Daily administration of the food protein is necessary to maintain the desensitization to the food allergen.

Systemic reactions are uncommon with SLIT and have only been reported in up to 2.3% of doses [49,50,51,52,53]. Overall, SLIT has fewer side effects when compared to OIT and symptoms are typically mild and localized to the oropharyngeal regions. EoE has not been observed with food allergen SLIT but has been reported in aeroallergen SLIT [54].

Clinical SLIT trials have been performed for cow’s milk [55,56], peanut [49,51,53], hazelnut [52], peach [50,56], and kiwi [57,58]. Overall, SLIT leads to an increased threshold of reactivity when taken regularly, however sustained unresponsiveness was minimal to none following a period of SLIT discontinuation [46,49,51,53,59]. It is important for patients to understand that like OIT, SLIT is not a cure and tolerance can be lost if there is a prolonged interruption in treatment.

## 5. Epicutaneous Immunotherapy

Unlike oral or SLIT, epicutaneous immunotherapy (EPIT) has provided an alternative route of desensitization to a food allergen by way of the skin. In EPIT, an adhesive patch is applied to the back or inner arm and worn for 24 h. Similar to OIT and SLIT, the dose must be administered daily in order to achieve and maintain desensitization to the food protein, however, the dose is fixed and significantly lower (250 μg) [60].

The safety profile for EPIT has so far been favorable. Mild local reactions at the patch site have been mainly observed in over 90% of patients receiving the active treatment (peanut EPIT) and mild non-local reactions were observed in less than 20% of subjects. Systemic reactions have not been reported with EPIT, as yet [60,61].

Earlier studies for cow’s milk EPIT suggested a trend toward cow’s milk desensitization, but the results of the study were limited by the small sample size and the findings were not statistically significant [62]. Investigations for peanut EPIT have been reported for phase 2 trials [60,61]. Recent findings from a phase 2b double blind placebo-controlled dose-ranging trial of the peanut EPIT demonstrated a response rate difference of 34.2% after 12 months of 250 μg peanut EPIT versus placebo in 6–11 year olds. The findings were not significant with the 50 or 100 μg dose or in older subjects [60]. Further results from phase 3 peanut EPIT trials are still ongoing. Phase 1 and 2 EPIT trials for cow’s milk are also underway.

## 6. Conclusions

New approaches for the management of food allergies are on the horizon and have demonstrated promising results. Overall, it is important for healthcare professionals to be aware of these new developments in the field of food allergy, in order to inform patients and their families about potential new treatment options.

Although OIT, SLIT, and EPIT treatments are not currently providing a cure for food allergy, when administered according to existing research protocols, protection from accidental food allergen exposure is observed in children who continue on regular therapy, whereas sustained unresponsiveness after discontinuation of treatment is much less common. It is currently unknown how long sustained unresponsiveness will persist after cessation of treatment. Overall, daily administration of treatment can provide protection from accidental exposures and lead to improved quality of life for patients and their families by reducing the fear and occurrence of accidental reactions. It is likely that food immunotherapy will be part of the mainstream management for food allergic children in the near future.

## Figures and Tables

**Table 1:** Outline of the different forms of food immunotherapy.

	Oral Immunotherapy	Sublingual Immunotherapy	Epicutaneous Immunotherapy
**Route of Administration**	Oral (mouth)	Under the tongue	On the skin
**Foods Evaluated**	Cow’s milk, hen’s egg, peanut, tree nuts, fruits, vegetables	Cow’s milk, peanut, hazelnut, kiwi	Cow’s milk, peanut
**Daily Doses** **(Food Protein)**	300–4000 mg	2–7 mg	100–500 μg
Efficacy *	Large	Small-to-Moderate	Variable
**Side Effects**	Common: local (oral or gastrointestinal)	Common: local (oral or pharyngeal)	Common: local (skin)
	Less common: systemic	Rare: systemic	Not yet reported: systemic

* Refers to desensitization effect, not sustained unresponsiveness (SUR).
